# The effect of SARS-CoV-2 variant on respiratory features and mortality

**DOI:** 10.1038/s41598-023-31761-y

**Published:** 2023-03-18

**Authors:** Thomas D. Hughes, Ajan Subramanian, Rana Chakraborty, Shannon A. Cotton, Maria Del Pilar Giraldo Herrera, Yong Huang, Natalie Lambert, Melissa D. Pinto, Amir M. Rahmani, Carmen Josefa Sierra, Charles A. Downs

**Affiliations:** 1 University of California, Irvine, Irvine, USA; 2 Mayo Clinic, Rochester, USA; 3 Indiana University, Bloomington, USA; 4 University of Miami, Coral Gables, USA

**Keywords:** Viral infection, Respiratory signs and symptoms

## Abstract

SARS-CoV-2 (COVID-19) has caused over 80 million infections 973,000 deaths in the United States, and mutations are linked to increased transmissibility. This study aimed to determine the effect of SARS-CoV-2 variants on respiratory features, mortality, and to determine the effect of vaccination status. A retrospective review of medical records (n = 55,406 unique patients) using the University of California Health COvid Research Data Set (UC CORDS) was performed to identify respiratory features, vaccination status, and mortality from 01/01/2020 to 04/26/2022. Variants were identified using the CDC data tracker. Increased odds of death were observed amongst unvaccinated individuals and fully vaccinated, partially vaccinated, or individuals who received any vaccination during multiple waves of the pandemic. Vaccination status was associated with survival and a decreased frequency of many respiratory features. More recent SARS-CoV-2 variants show a reduction in lower respiratory tract features with an increase in upper respiratory tract features. Being fully vaccinated results in fewer respiratory features and higher odds of survival, supporting vaccination in preventing morbidity and mortality from COVID-19.

## Introduction

The coronavirus disease of 2019 (COVID-19) pandemic caused by the novel SARS-CoV-2 virus was present on the west coast of the United States in either late 2019 or early 2020^1^. Since, there have been multiple waves of infection and ongoing viral mutations. According to the United States Centers for Disease Control and Prevention (CDC), as of April 2022, approximately 80 million people have contracted SARS-CoV-2 and 973,000 people have died^2^. In California alone, as of April 2022, there were more than 9,200,000 cases and 89,582 deaths^2^. The initial virus, termed the Founder variant, has mutated multiple times into other variants namely Alpha, Delta, and Omicron. With each mutation, the virus has become increasingly transmittable, and associated with a reduction in hospitalizations and fewer severe lower respiratory tract issues, the primary cause of COVID-19 deaths, compared to the Delta variant^2^.

Despite virus mutation, vaccination remains the best protection against hospitalization, death, and lessens the risk for post-covid conditions or post-acute sequelae of SARS-CoV-2 infection (i.e. long-COVID), which is defined as ongoing COVID-19 symptoms beyond the usual duration of acute disease^3–5^. According to the World Health Organization, approximately 63% of the US population is fully vaccinated^6^. Similarly, 66–67% of the population in the United Kingdom, Canada, and Australia are fully vaccinated^6^. There are several reasons why vaccinate uptake in the US is low. Barriers to vaccination can be structural or attitudinal^7^. According to Fisk^7^, structural barriers relate to access issues (e.g. cost, convenience, and supply chain issues), whereas attitudinal barriers are associated with low perceived risk of acquiring the disease or potential severe consequences from the disease, perceived risks of the vaccine, lack of trust of agencies that are responsible for the development and distribution of vaccines, misinformation, or misconceptions^7^. Therefore, ongoing research is necessary to confront the attitudinal barriers members of the population may have to promote increased vaccine uptake to the target of 70–90% to achieve herd immunity and thus prevent and mitigate the ongoing significant morbidity and mortality, disruptions to society, and long-term health consequences^8^.

Severe COVID-19 infection may lead to viral pneumonia and acute respiratory distress syndrome (ARDS). One study by Wu et al.^9^ found that 42% of patients with COVID-19 pneumonia developed ARDS^9^. In addition to acute complications such as ARDS, a recent publication from the National Health Service in the United Kingdom suggested that chronic cough, respiratory fatigue, and fibrotic lung disease complicate long-term recovery from COVID-19^10^. The National Health Service^11^ examined long-term symptoms of diseases caused by other coronaviruses, such as severe acute respiratory syndrome (SARS) and Middle East respiratory syndrome (MERS) and found that up to 30% of patients had persistent lung abnormalities after recovering from the acute illness stage^11^. Furthermore, a study by Huang et al.^12^ linked respiratory symptom clusters with a higher risk of long-term COVID-19 or long-haul COVID-19^12^. Collectively, these studies illustrate the importance of the respiratory system in SARS-CoV-2 infection and subsequent COVID-19 diseases.

The purpose of this study was to assess the prevalence of upper and lower respiratory tract symptoms across SARS-CoV-2 variants, to determine the effect of vaccination status on the symptoms, and to evaluate the risks of mortality based on variant type and vaccination status. A retrospective review of 55,406 medical records from hospitalized patients with confirmed SARS-CoV-2 infection within the University of California Health Covid Research Data Set (UC CORDS) was performed.

## Methods

### University of California health covid research data set (UC CORDS)

The UC CORDS data set comprises de-identified health data across all facilities in the University of California (UC) Health system, encompassing 19 health professional schools, five academic medical centers, and 12 hospitals^13^. It contains the records of more than 700,000 patients, including those hospitalized and outpatient, with de-identified information to enable safe and secure clinical research. The data in the UC CORDS database is stored in an online enclave and is not available to the general public, only those with access granted by the University of California may access UC CORDS. This study was deemed exempted from obtaining ethical approval by the University of California, Irvine, Internal Review Board. All methods were carried out in accordance with relevant university guidelines and regulations. The UC CORDS data set contains de-identified data from individuals seeking care in the University of California Health System, as such the Institutional Review Board at the University of California, Irvine, waived the need for obtaining informed consent. No experimental protocols were used in this study of de-identified data; therefore, no approvals were sought from the University.

### Variant and vaccination status

The UC CORDS data set did not report variant type; therefore, variants were identified based upon dates when each variant was dominant as reported by the CDC data tracker^2^. Although the CDC data tracker contains national-level data, the data from the California Department of Public Health in daily trends of the number of COVID-19 cases was not remarkably different to warrant focusing on California-specific data, as shown in the Supplementary Information Figs. 1 and 2^2,14^. Moreover, this method was also previously used by Wang et al.^3^ to classify COVID-19 patient in their analysis of outcomes for the US population; although it may lack the precision of including variant-confirmed speciation from laboratory testing, it is nonetheless a measure that reflects the dominant variant waves that infected the US population at ongoing timepoints throughout the pandemic^3^. Accordingly, the date ranges extended from 01/01/2020 to 06/30/2020 for the Founder variant, 06/30/2020 to 05/31/2021 for the Alpha variant, 06/01/2021 to 11/30/2021 for the Delta variant, and from 12/01/2021 to 04/26/2022 for the Omicron variant. Although vaccines were not available for COVID-19 infection until December 2020, SARS-CoV-2 variants were included in this study prior to the vaccine being available were included primarily to depict the evolutionary changes in symptom presentation over time with each variant, rather than to focus on a comparison of fully vaccinated, partially vaccinated, or unvaccinated status. Patients who received at least two doses of the vaccine before their positive test result were considered fully vaccinated. Patients who received one dose of the vaccine before their positive test result were considered partially vaccinated, and those who received no vaccine were considered unvaccinated.

### Inclusion and exclusion criteria and sample

The study involved review of electronic health record data in the UC CORDS data set. Inclusion criteria for this study included all patients, regardless of age, who had a positive test anywhere in the hospital setting (i.e. emergency department, intensive care unit, or any other hospital unit). For any given positive test, respiratory features data was included in a window of 5 days prior to the test result and up to 30 days after a positive RT-PCR test for SARS-CoV-2. Exclusion criteria for the study included those whose data was obtained from non-hospital (i.e. clinic-based) outpatient settings and those who had a positive RT-PCR SARS-CoV-2 test outside of the predetermined window.

Demographic data were obtained by searching the data set for each demographic variable of interest, including age, gender, and race/ethnicity data. Race and ethnicity data were included to ensure the data set included a representational sample of the general population in California. Comorbidity data was obtained by searching the data set for the 200 most common ICD-10 codes listed for the patients and then filtered further by including duplicate terms (e.g. “chronic obstructive pulmonary disease (COPD)” and “COPD”) and removing terms that were not pertinent to this study (e.g. pregnancy).

### Respiratory feature identification and extraction

The 40 most reported features across all body systems were extracted from each variant through a query in the UC CORDS data set. Forty features were chosen as an initial starting point for assessing the number of features based on historical work that found features may range from as few as 17^15^ to as many as 50 features^16^. In this study, the total number of features per variant ranged from 27 features during the Founder wave and up to 34 features during the Delta variant. Therefore, there were no remaining features undiscovered from the search results.

The preliminary search to identify prominent respiratory features involved running a query for the top 40 most frequent ICD-10 codes amongst all the patients in the data set. The term “features” was substituted for ICD-10 codes to account for the differing nature of the results; for example, some ICD-10 codes are medical diagnoses, (e.g. acute respiratory failure or pneumonia) while others may be symptoms that a patient reports (e.g. cough or nasal congestion), while others may be signs that a medical provider assesses (e.g. dyspnea). The feature selection was then compared with historical work by others which demonstrated the most prevalent signs and symptoms (i.e. “features”) that affected people with acute and post-acute SARS-CoV-2 infection^15,16^ to assess face validity and consistency with prior work of the retrieved data. Of these most reported features across each variant, the non-respiratory features were classified as “unclassified” and the remaining respiratory features were assigned classification into lower and upper respiratory features through expert consultation and discussion amongst the research group. All features, their ranking, and their classifications are provided in a tabular format in Supplementary Information Tables 1, 2, 3 and 4. The reported frequency of each feature was then normalized per 100 cases.

### Statistical analysis

Statistical analysis was performed using odds ratios to determine the risk of death for each variant while accounting for vaccination status and adjusted odds ratios were calculated while controlling for age and gender. In this study, age was represented as a continuous variable and the reference category for gender was men. The two-tailed Chi-square test was used to study the effect of respiratory symptoms of COVID-19 on vaccination status across variants. Chi-square tests are used to study the relationship between categorical variables using a contingency matrix. The tests compared the relationship between the frequency of patients who did and did not report a particular symptom. The contingency matrices were created for each variant separately and they each compared the frequency of a particular symptom between fully and not fully vaccinated patients. A *p*-value of < 0.05 was considered statistically significant. Analyses were conducted using Python (version 3.6) and the SciPy package (version 1.8.0). Because group sizes between the fully vaccinated and partially vaccinated were disproportionate, balancing of the groups was done using the SKlearn (version 1.1.3) package. This was done by randomly sampling patients from the larger group to match the size of the smaller group. This subsampled data was then placed into a logistic regression model using the SKlearn package with age and gender as covariates to obtain adjusted odds ratios for mortality.

## Results

### Demographics and comorbidities

Tables 1, 2, 3 and 4 provide demographic information for the 55,406 patients included based on variant and vaccine status (Founder (n = 2319), Alpha (n = 16,753), Delta (n = 7280), and Omicron (n = 29,054)). Across all variants, the fully vaccinated population was the oldest, followed by the partially vaccinated, and finally with the unvaccinated population being the youngest. Additionally, each of the major variant waves included more females than males, and the sample was predominantly White, followed by Hispanic or Latino, and then Asian, African-American, Native Hawaiian or Pacific Islander, American Indian or Alaskan Native, and Unknown or other.

**Table 1:** Demographic information of patients with a positive SARS-CoV-2 result during the Founder variant wave (n = 2319 SARS-CoV-2 infections).

	Fully vaccinated (n = 0)	Partially vaccinated (n = 0)	Unvaccinated (n = 2319)
Age, years (mean)	–	–	46.59 ± 20.18
Gender			
Female (n, %)	–	–	1126 (48.6)
Male (n, %)	–	–	1193 (51.4)
Unknown or other (n, %)	–	–	0
Race			
White (n, %)	–	–	638 (27.5)
Hispanic or Latino (n, %)	–	–	1050 (45.27)
Asian (n, %)	–	–	157 (6.8)
African American (n, %)	–	–	118 (5.1)
Native Hawaiian or Pacific Islander (n, %)	–	–	15 (0.6)
American Indian or Alaskan native (n, %)	–	–	6 (0.3)
Unknown or other (n, %)	–	–	335 (14.4)

**Table 2:** Demographic information of patients with a positive SARS-CoV-2 result during the Alpha variant wave (n = 16,753 SARS-CoV-2 infections).

	Fully vaccinated (n = 95)	Partially vaccinated (n = 343)	Unvaccinated (n = 16,315)
Age, years (mean)	63.77 ± 17.38	54.57 ± 19.16	44.25 ± 21.67
Gender			
Female (n, %)	45 (47.4)	204 (59.5)	8232 (50.4)
Male (n, %)	50 (52.6)	139 (40.5)	8081 (49.5)
Unknown or other (n, %)	0	0	2 (0.01)
Race			
White (n, %)	44 (46.3)	116 (33.8)	5047 (30.9)
Hispanic or Latino (n, %)	24 (25.3)	107 (31.2)	6407 (39.3)
Asian (n, %)	11 (11.6)	55 (16.0)	1008 (6.2)
African American (n, %)	4 (4.2)	16 (4.7)	984 (6.0)
Native Hawaiian or Pacific Islander (n, %)	1 (1.0)	6 (1.8)	99 (0.6)
American Indian or Alaskan native (n, %)	0 (0)	0 (0)	33 (0.2)
Unknown or Other (n, %)	11 (11.6)	43 (12.5)	2737 (16.7)

**Table 3:** Demographic information of patients with a positive SARS-CoV-2 result during the Delta variant wave (n = 7280 SARS-CoV-2 infections).

	Fully vaccinated (n = 1542)	Partially vaccinated (n = 493)	Unvaccinated (n = 5245)
Age, years (mean)	52.4 ± 18.31	45.57 ± 16.71	36.78 ± 21.69
Gender			
Female (n, %)	823 (53.4)	256 (51.9)	2674 (50.9)
Male (n, %)	719 (46.6)	234 (47.6)	2569 (48.9)
Unknown or other (n, %)	0	3 (0.6)	2 (0.03)
Race			
White (n, %)	827 (53.4)	198 (40.2)	1993 (37.9)
Hispanic or Latino (n, %)	304 (19.7)	122 (24.7)	1400 (26.7)
Asian (n, %)	140 (9.1)	49 (9.9)	317 (6.0)
African American (n, %)	69 (4.5)	33 (6.7)	474 (9.0)
Native Hawaiian or Pacific Islander (n, %)	14 (0.9)	5 (1.0)	29 (0.5)
American Indian or Alaskan native (n, %)	2 (0.1)	4 (0.8)	19 (0.4)
Unknown or other (n, %)	186 (12.1)	82 (16.6)	1013 (19.3)

**Table 4:** Demographic information of patients with a positive SARS-CoV-2 result during the Omicron variant wave (n = 29,054 SARS-CoV-2 infections).

	Fully vaccinated (n = 9919)	Partially vaccinated (n = 3371)	Unvaccinated (n = 15,764)
Age, years (mean)	46.79 ± 19.34	45.12 ± 17.25	34.12 ± 23.53
Gender			
Female (n, %)	5721 (57.7)	1999 (59.3)	8342 (52.9)
Male (n, %)	4168 (42.0)	1366 (40.5)	7400 (46.9)
Unknown or other (n, %)	30 (0.3)	6 (0.18)	1 (0.01)
Race			
White (n, %)	4245 (42.8)	1349 (40.0)	5046 (32)
Hispanic or Latino (n, %)	2294 (23.1)	745 (22.1)	4367 (27.7)
Asian (n, %)	1309 (13.2)	580 (17.2)	1483 (9.4)
African American (n, %)	599 (6.04)	190 (5.6)	1240 (7.9)
Native Hawaiian or Pacific Islander (n, %)	93 (0.9)	29 (0.9)	96 (0.6)
American Indian or Alaskan native (n, %)	19 (0.19)	11 (0.3)	39 (0.2)
Unknown or other (n, %)	1360 (13.7)	467 (13.9)	3493 (22.2)

Tables 5, 6, 7 and 8 provide patient comorbidity data delineated by variant. Across all variants, the fully vaccinated population had a higher frequency of chronic conditions such as anemia, atrial fibrillation, COPD, cancer, gastrointestinal reflux disease (GERD), heart failure, hypertension, immunocompromised, kidney disease, obesity, and type 2 diabetes mellitus.

**Table 5:** Comorbidity information of patients with a positive SARS-CoV-2 result during the Founder variant wave (n = 2319 SARS-CoV-2 infections) expressed as frequency percentage.

Condition	Fully vaccinated (%)	Partially vaccinated (%)	Unvaccinated (%)
AIDS	–	–	1.63
Anemia	–	–	9.16
Anxiety/depression	–	–	43.38
Asthma	–	–	7.24
Atrial fibrillation	–	–	9.29
COPD	–	–	3.26
Cancer	–	–	5.59
Cardiomyopathy	–	–	5.59
Dementia	–	–	1.16
GERD	–	–	3.34
Heart failure	–	–	21.92
Hyperlipidemia	–	–	14.04
Hypertension	–	–	28.52
Immunocompromised	–	–	2.87
Kidney disease	–	–	34.3
Liver disease	–	–	2.74
Myocardial infarction	–	–	0
Obesity	–	–	20.04
Peripheral vascular disease	–	–	1.71
Rheumatoid arthritis	–	–	1.03
SLE	–	–	0.6
Transient ischemic attack	–	–	0.04
Transplant	–	–	1.71
Type 1 diabetes mellitus	–	–	0.6
Type 2 diabetes mellitus	–	–	26.72

**Table 6:** Comorbidity information of patients with a positive SARS-CoV-2 result during the Alpha variant wave (n = 16,753 SARS-CoV-2 infections) expressed as frequency percentage.

Condition	Fully vaccinated (%)	Partially vaccinated (%)	Unvaccinated (%)
AIDS	0	1.16	0.89
Anemia	21.05	15.66	10.04
Anxiety/depression	64.21	62.32	44.47
Asthma	10.53	8.7	8.45
Atrial fibrillation	33.68	14.5	8.54
COPD	1.05	6.67	4.54
Cancer	17.89	9.57	6.89
Cardiomyopathy	4.21	4.64	2.28
Dementia	4.21	4.64	2.27
GERD	50.53	39.42	24.54
Heart failure	64.21	32.48	16.44
Hyperlipidemia	30.53	25.21	15.51
Hypertension	53.68	37.39	28.26
Immunocompromised	20	6.67	4
Kidney disease	86.31	44.07	31.77
Liver disease	10.53	4.35	3.49
Myocardial infarction	1.05	0	0.02
Obesity	22.11	24.64	21.82
Peripheral vascular disease	7.37	2.9	2.01
Rheumatoid arthritis	0	0.87	1.13
SLE	1.05	0.87	0.68
Transient ischemic attack	0	0	0.05
Transplant	12.63	3.19	2.34
Type 1 diabetes mellitus	1.05	0.58	0.67
Type 2 diabetes mellitus	40	33.62	25.34

**Table 7:** Comorbidity information of patients with a positive SARS-CoV-2 result during the Delta variant wave (n = 7280 SARS-CoV-2 infections) expressed as frequency percentage.

Condition	Fully vaccinated (%)	Partially vaccinated (%)	Unvaccinated (%)
AIDS	2.4	3.83	0.5
Anemia	15.13	8.67	7.4
Anxiety/depression	57.92	63.32	37.9
Asthma	9.94	7.06	8.55
Atrial fibrillation	14.61	7.05	4.97
COPD	8.57	5.04	5.62
Cancer	12.55	5.63	4.97
Cardiomyopathy	3.11	1.41	1.54
Dementia	1.17	0.6	0.62
GERD	34.39	27.62	17.74
Heart failure	22.46	15.33	8.85
Hyperlipidemia	22.99	14.72	10.9
Hypertension	36.3	23.79	18.97
Immunocompromised	10.26	7.06	3.5
Kidney disease	44.74	28.64	18.53
Liver disease	3.96	2.22	1.87
Myocardial infarction	0	0	0.02
Obesity	22.01	19.96	15.84
Peripheral vascular disease	3.05	1.41	1.17
Rheumatoid arthritis	1.75	1.41	0.69
SLE	1.36	0.2	0.48
Transient ischemic attack	0.06	0	0.02
Transplant	7.34	3.63	1.79
Type 1 diabetes mellitus	1.23	1.41	0.75
Type 2 diabetes mellitus	26.24	19.15	13.79

**Table 8:** Comorbidity information of patients with a positive SARS-CoV-2 result during the Omicron variant wave (n = 29,054 SARS-CoV-2 infections) expressed as frequency percentage.

Condition	Fully vaccinated (%)	Partially vaccinated (%)	Unvaccinated (%)
AIDS	1.71	2.1	0.54
Anemia	12.87	9.45	7.44
Anxiety/depression	57.94	50.73	39.3
Asthma	9.04	8.98	7.99
Atrial fibrillation	8.7	6.9	4.55
COPD	6.54	5.04	3.56
Cancer	9.36	6.04	5.73
Cardiomyopathy	2.64	1.92	1.41
Dementia	1.23	0.62	0.69
GERD	31.37	26.81	21.7
Heart failure	15.74	12.75	7.99
Hyperlipidemia	19.41	16.53	12.17
Hypertension	29.93	25.21	18.65
Immunocompromised	7.56	4.71	3.59
Kidney disease	33.57	24.65	16.84
Liver disease	2.96	2.46	1.85
Myocardial infarction	0.06	0	0.01
Obesity	20.67	18.63	13.9
Peripheral vascular disease	2.3	1.69	1.05
Rheumatoid arthritis	1.66	1.01	0.82
SLE	0.93	0.8	0.66
Transient ischemic attack	0.04	0.06	0.01
Transplant	4.9	3.17	1.8
Type 1 diabetes mellitus	0.94	0.8	0.56
Type 2 diabetes mellitus	22.74	17.69	12.6

### Respiratory feature frequency

**Figure 1:**
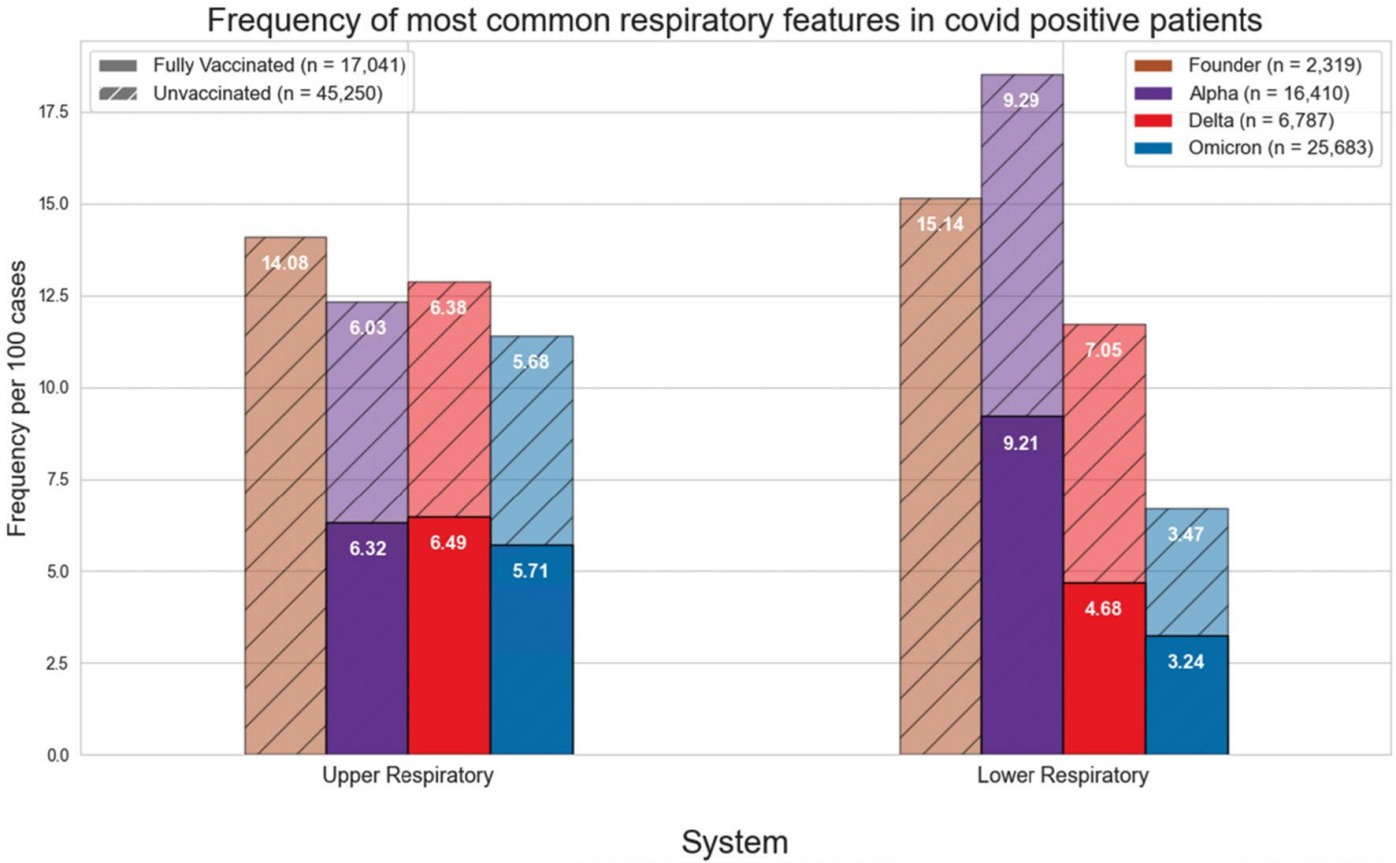
Comparison of frequency of most common respiratory features in COVID positive patients based on fully vaccinated or unvaccinated status.

**Figure 2:**
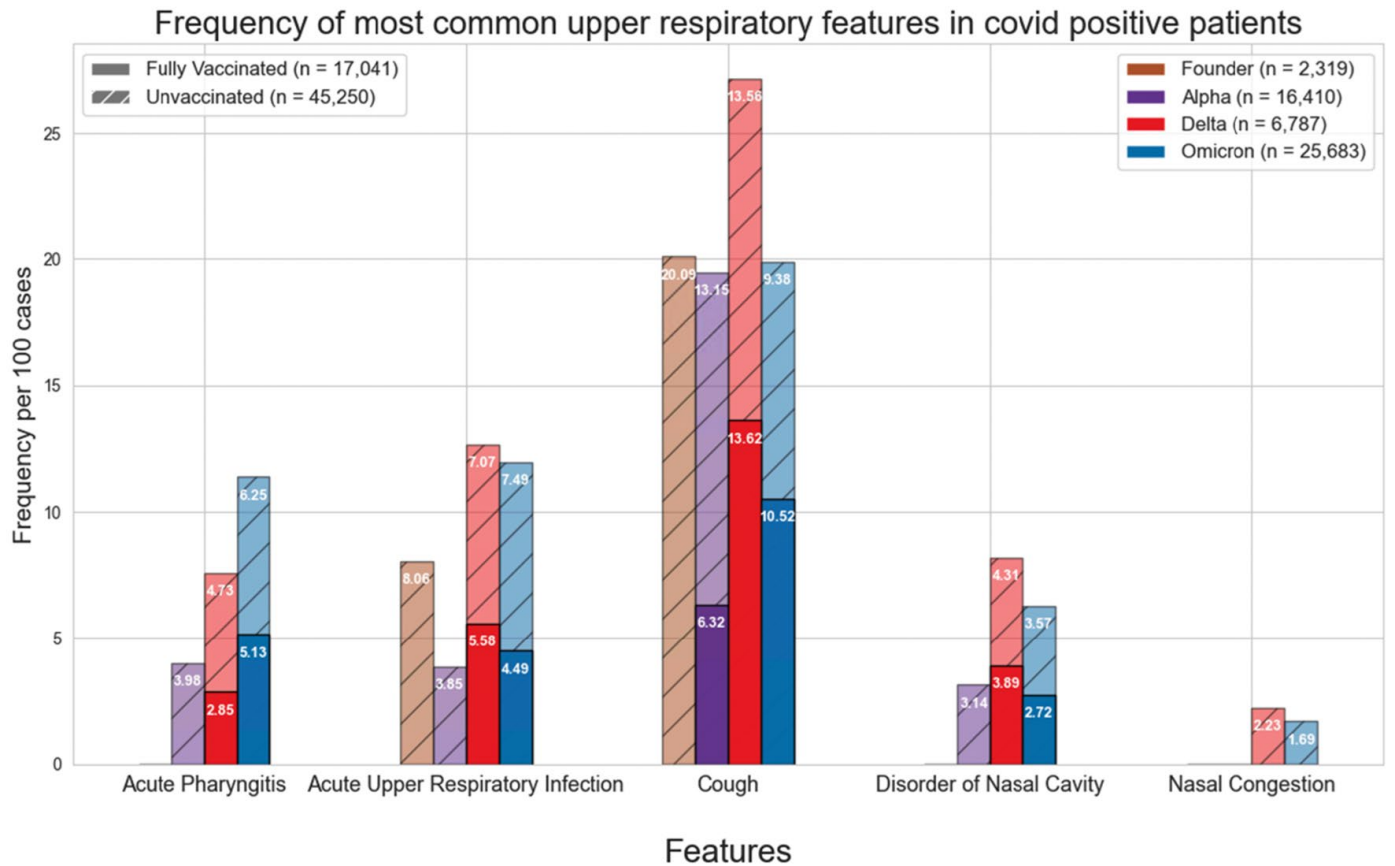
Comparison of frequency of most common upper respiratory features in COVID positive patients based on fully vaccinated or unvaccinated status.

**Figure 3:**
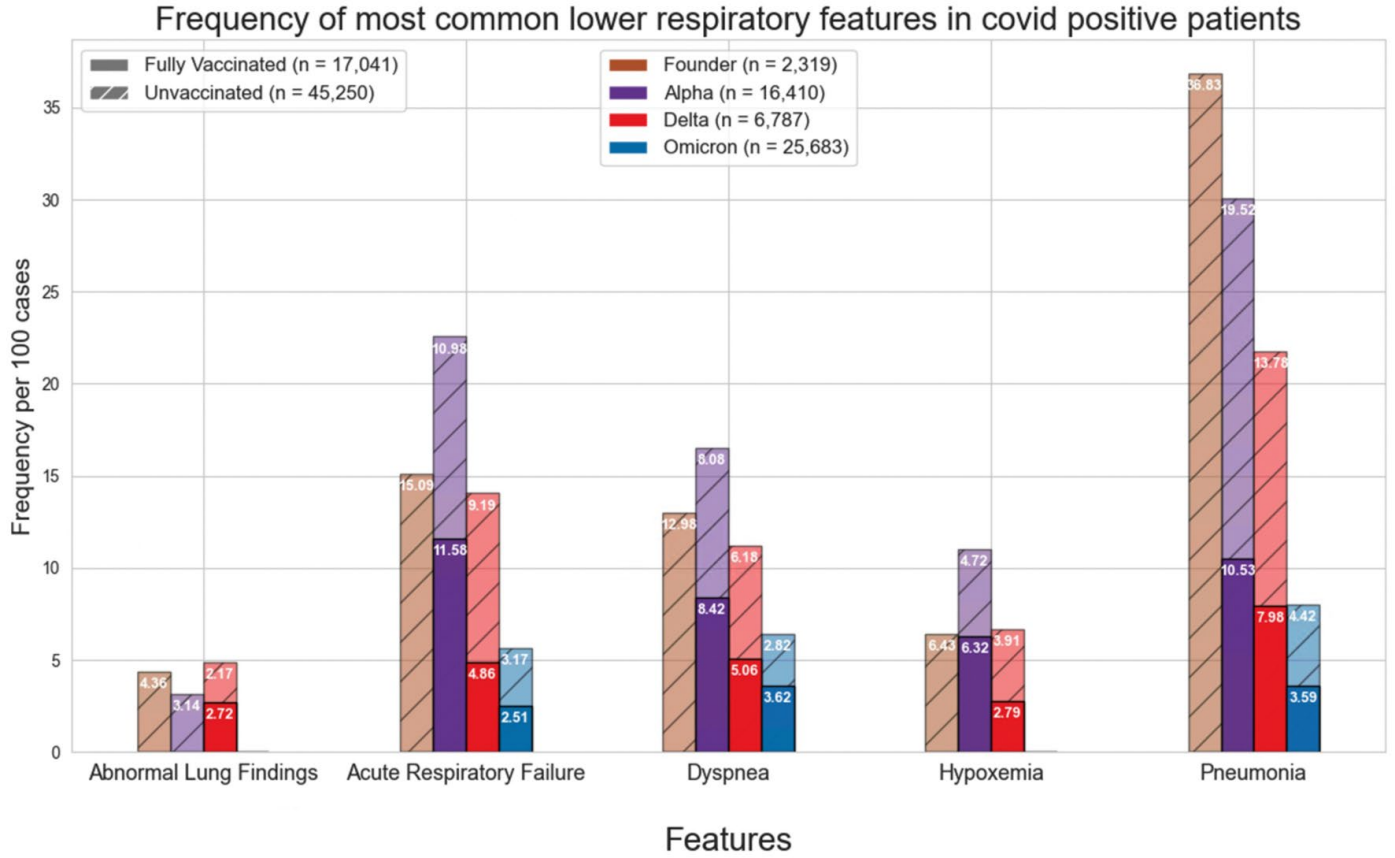
Comparison of frequency of most common lower respiratory features in COVID positive patients based on fully vaccinated or unvaccinated status.

**Figure 4:**
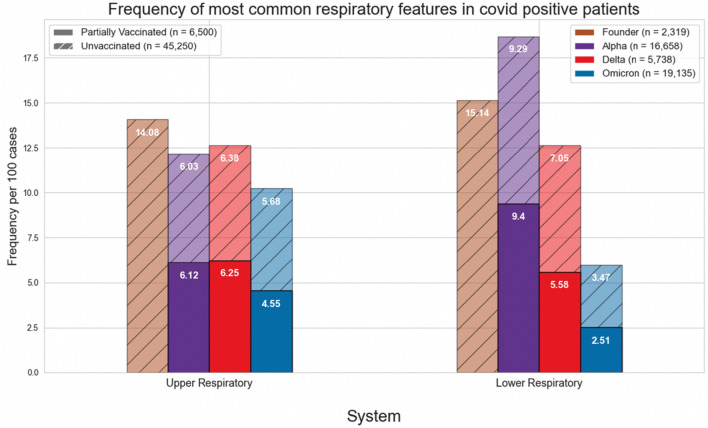
Comparison of frequency of most common respiratory features in COVID positive patients based on partially vaccinated or unvaccinated status.

**Figure 5:**
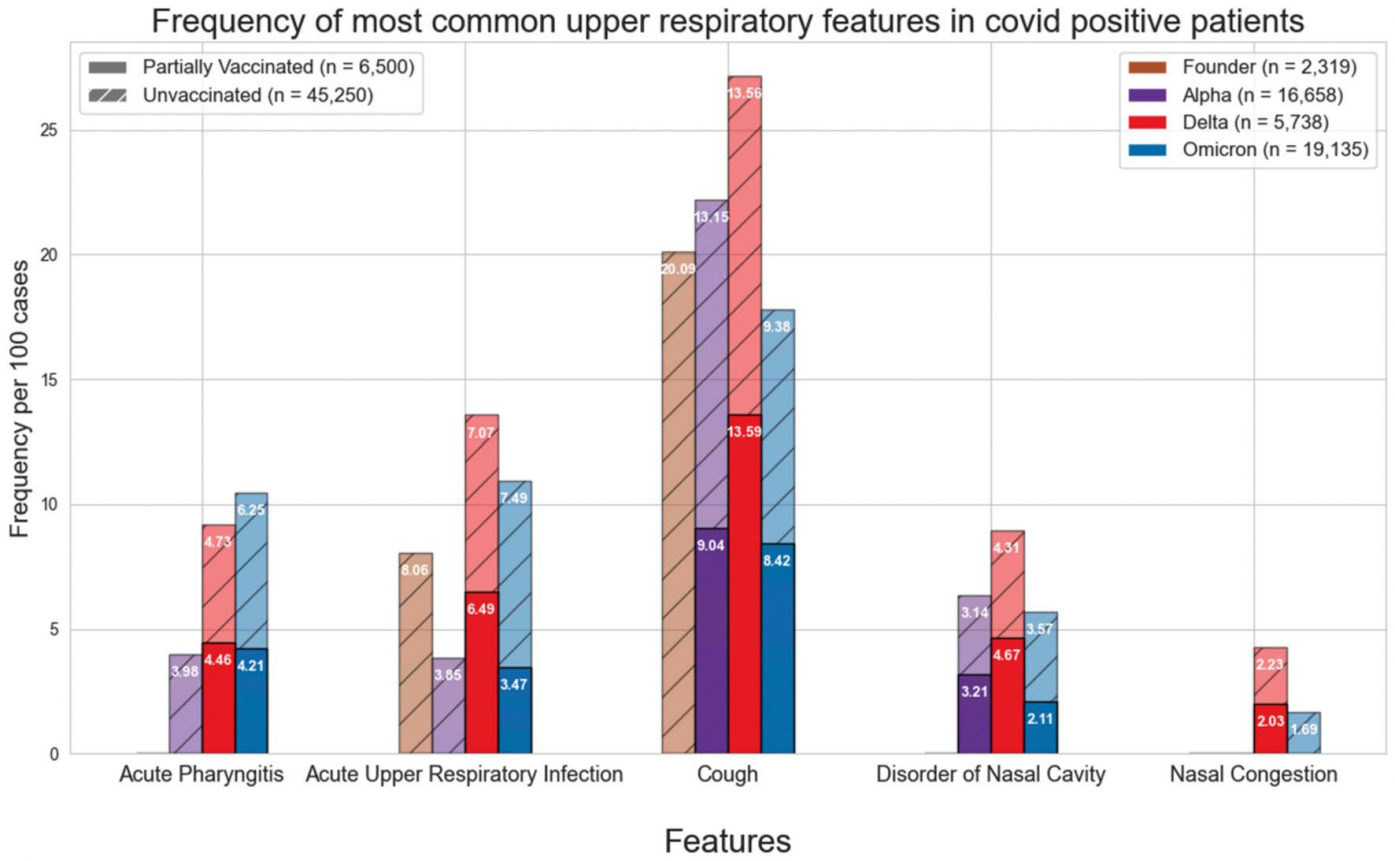
Comparison of frequency of most common upper respiratory features in COVID positive patients based on partially vaccinated or unvaccinated status.

**Figure 6:**
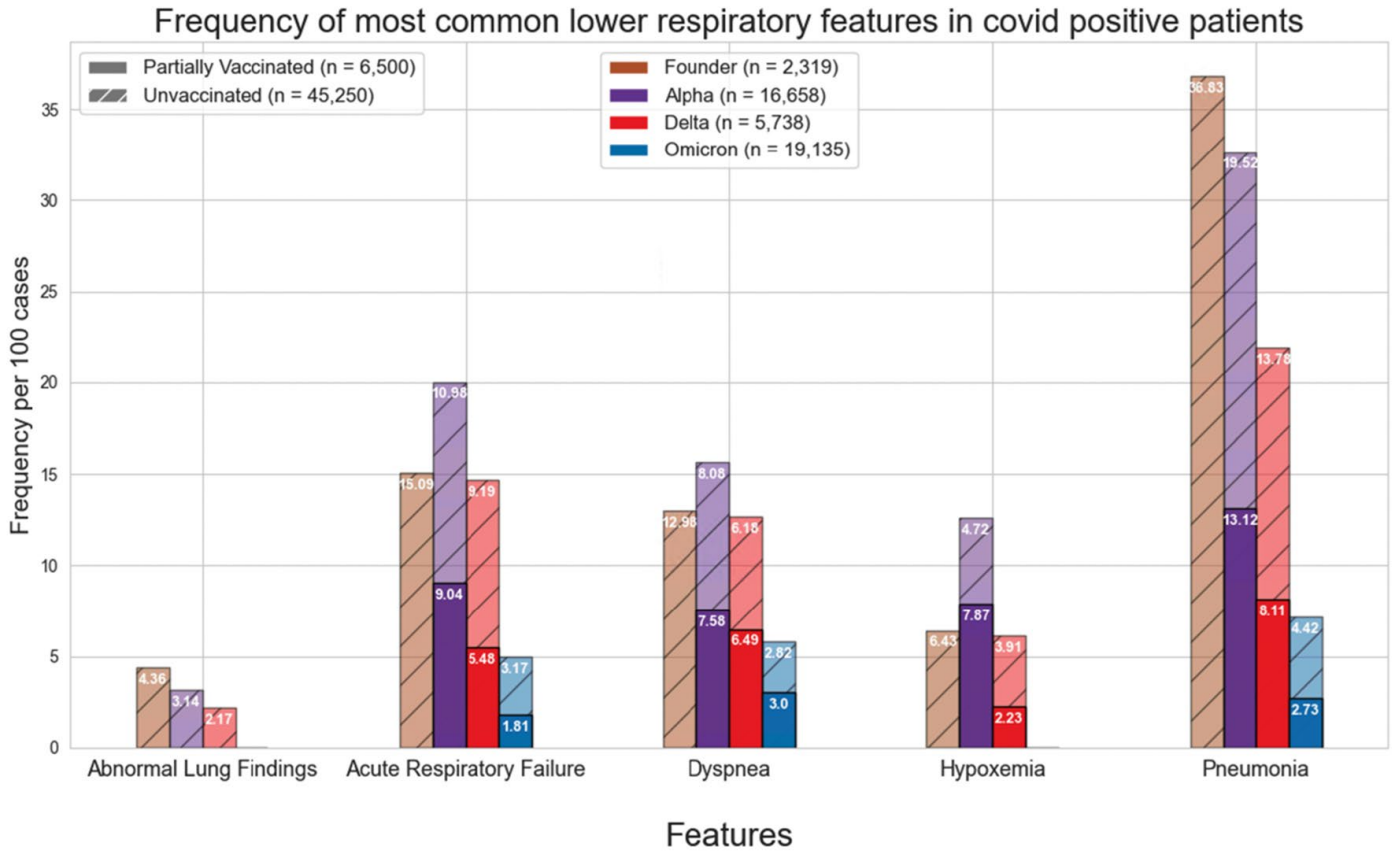
Comparison of frequency of most common lower respiratory features in COVID positive patients based on partially vaccinated or unvaccinated status.

Figures 1 and 4 show the normalized cases of aggregated respiratory features, upper respiratory features, or lower respiratory tract features based on the variant and vaccination status. Upper respiratory tract features included acute pharyngitis, acute upper respiratory tract infection, cough, disorder of nasal cavity, and nasal congestion. Lower respiratory tract features included abnormal lung findings, acute respiratory failure, dyspnea, hypoxemia, and pneumonia. Figures 2, 3, 5 and 6 show the normalized cases of upper and lower respiratory features based on variant and vaccination status, respectively. Of note, the frequency of lower respiratory tract features decreased with successive variants while upper respiratory tract features increased.

### Mortality

Tables 9, 10 and 11 provide mortality data based on vaccination status for all patients in the study across the four major variants. Table 9, which compares mortality data between fully vaccinated and unvaccinated individuals, shows that there was not a statistically significant difference between the groups regarding mortality in the unadjusted analysis. However, in the adjusted analysis, the odds of death for unvaccinated individuals reached significance during the Delta and Omicron waves. During the Delta wave, the adjusted odds ratio of mortality for unvaccinated individuals was 1.21; during the Omicron wave, the adjusted odds ratio of mortality for unvaccinated individuals was 1.17. Additionally, there were substantially more patients in the unvaccinated group (n = 1344) than the fully vaccinated group (n = 158).

**Table 9:** Comparison of mortality rate and mortality odds ratio by variants between fully vaccinated and unvaccinated individuals.

	Fully vaccinated (n = 158)	Unvaccinated (n = 1344)	Odds ratio	Adjusted odds ratio
Founder (n, %)	N/A	87 (3.82)	N/A	N/A
Alpha (n, %)	8 (7.8)	934 (5.95)	0.74	2.13
Delta (n, %)	29 (1.89)	135 (2.61)	1.39	1.21*
Omicron (n, %)	121 (1.24)	188 (1.19)	0.95	1.17*

*p*-value < 0.05 is significant, *denotes *p*-value < 0.05.

**Table 10:** Comparison of mortality rate and mortality odds ratio by variants between partially vaccinated and unvaccinated individuals.

	Partially vaccinated (n = 51)	Unvaccinated (n = 1344)	Odds ratio	Adjusted odds ratio
Founder (n, %)	N/A	87 (3.82)	N/A	N/A
Alpha (n, %)	11 (3.09)	934 (5.95)	1.98*	1.93
Delta (n, %)	4 (0.81)	135 (2.61)	3.26*	1.23
Omicron (n, %)	36 (1.28)	188 (1.19)	0.92	1.22

*p*-value < 0.05 is significant, *denotes *p*-value < 0.05.

**Table 11:** Comparison of mortality rate and mortality odds ratio by variants between fully or partially individuals and unvaccinated individuals.

	Fully or partially vaccinated (n = 209)	Unvaccinated (n = 1344)	Odds ratio	Adjusted odds ratio
Founder (n, %)	N/A	87 (3.82)	N/A	N/A
Alpha (n, %)	19 (4.15)	934 (5.95)	1.46	1.73
Delta (n, %)	33 (1.63)	135 (2.61)	1.62*	1.17*
Omicron (n, %)	157 (1.25)	188 (1.19)	0.95	1.20*

*p*-value < 0.05 is significant, *denotes *p*-value < 0.05.

Table 10 shows mortality data across all variants based on partially vaccinated or unvaccinated status. In the unadjusted analysis, during the Alpha and Delta waves, unvaccinated individuals had a significantly higher likelihood of mortality compared with partially vaccinated individuals (Alpha OR: 1.98, *p* = 0.022; Delta OR: 3.26, *p* = 0.009). However, in the adjusted analysis, there were no statistically significant differences in mortality between partially vaccinated and unvaccinated individuals. Similarly, there were substantially more individuals in the unvaccinated group (n = 1381) compared with the partially vaccinated group (n = 51).

Table 11 shows the mortality data across all variants between individuals who were either fully vaccinated or partially vaccinated compared to unvaccinated individuals. In the unadjusted analysis, during the Delta wave, unvaccinated individuals had higher risk of death compared to individuals who were either partially or fully vaccinated (Delta OR: 1.62, *p* = 0.012). In the adjusted analysis, unvaccinated individuals were significantly more likely to die compared with individuals who received any vaccination (Delta adjusted OR: 1.17, *p* = 0.024; Omicron adjusted OR: 1.2, *p* = 0.003). Again, there were a higher number of individuals in unvaccinated group (n = 1344) than either the partially or fully vaccinated group (n = 209).

### Effect of vaccination status

The effect of vaccine status on respiratory features for each variant wave was assessed in Tables 12, 13 and 14. Across all variants, a total of 11,556 individuals were fully vaccinated, 4207 were partially vaccinated, and 39,646 were unvaccinated. Tables 12 and 13 show that unvaccinated individuals have an increased odds of having many upper and lower respiratory features. Also, Table 14 shows that unvaccinated individuals have increased odds of many upper and lower respiratory features compared with individuals who received any vaccination.

**Table 12:** Chi-square tests comparing feature significance in fully vaccinated versus unvaccinated individuals across the Alpha, Delta and Omicron waves.

Variant	Feature	Fully vaccinated (n = 11,556)	Unvaccinated (n = 39,646)	Odds ratio	Chi-square *p*-value
Alpha	Pneumonia	12	3648	1.99	0.032
Delta	Acute pharyngitis	44	248	1.69	< 0.01
	Acute respiratory failure	88	577	2.04	< 0.001
	Acute upper respiratory infection	86	371	1.28	0.045
	Hypoxemia	43	205	1.42	0.047
	Pneumonia	161	835	1.62	< 0.001
Omicron	Abnormal lung findings	128	127	0.62	< 0.001
	Acute pharyngitis	508	984	1.23	< 0.001
	Acute respiratory failure	287	604	1.34	< 0.001
	Acute upper respiratory infection	445	1176	1.72	< 0.001
	Cough	1043	1477	0.88	< 0.01
	Disorder of nasal cavity	270	561	1.32	< 0.001
	Dyspnea	359	444	0.77	< 0.001
	Pneumonia	452	891	1.255	< 0.001

*p*-value < 0.05 is significant.

**Table 13:** Chi-square tests comparing feature significance in partially vaccinated versus unvaccinated individuals across the Alpha, Delta and Omicron waves.

Variant	Feature	Partially vaccinated (n = 4207)	Unvaccinated (n = 39,646)	Odds ratio	Chi-square *p*-value
Alpha	Cough	31	2145	1.52	0.031
	Hypoxemia	27	770	0.58	< 0.01
	Pneumonia	56	3648	1.48	< 0.01
Delta	Acute respiratory failure	34	577	1.67	< 0.01
	Pneumonia	45	835	1.89	< 0.001
Omicron	Abnormal lung findings	40	127	0.68	0.04
	Acute pharyngitis	141	984	1.53	< 0.001
	Acute respiratory failure	73	604	1.802	< 0.001
	Acute upper respiratory infection	116	1176	2.26	< 0.001
	Disorder of nasal cavity	71	561	1.717	< 0.001
	Hypoxemia	19	234	2.66	< 0.001
	Nasal congestion	28	266	2.05	< 0.001
	Pneumonia	129	891	1.51	< 0.001

*p*-value < 0.05 is significant.

**Table 14:** Chi-square tests comparing feature significance in any vaccinated versus unvaccinated individuals across the Alpha, Delta and Omicron waves.

Variant	Feature	Fully or partially vaccinated (n = 15,763)	Unvaccinated (n = 39,646)	Odds ratio	Chi-square *p*-value
Alpha	Acute upper respiratory infection	8	628	2.15	0.039
	Cough	37	2145	1.64	< 0.01
	Hypoxemia	33	770	0.61	< 0.01
	Pneumonia	68	3648	1.57	< 0.001
Delta	Acute pharyngitis	66	248	1.48	< 0.01
	Acute respiratory failure	122	577	1.94	< 0.001
	Hypoxemia	54	205	1.49	0.012
	Pneumonia	206	835	1.68	< 0.001
Omicron	Abnormal lung findings	168	127	0.63	< 0.001
	Acute pharyngitis	649	984	1.29	< 0.001
	Acute respiratory failure	360	604	1.43	< 0.001
	Acute upper respiratory infection	561	1176	1.83	< 0.001
	Disorder of nasal cavity	341	561	1.40	< 0.001
	Dyspnea	460	444	0.81	< 0.01
	Nasal congestion	173	266	1.30	< 0.01
	Pneumonia	581	891	1.31	< 0.001

*p*-value < 0.05 is significant.

## Discussion

Acute COVID-19 infection causes numerous respiratory disorders, and as such, it is necessary to investigate its impacts across the respiratory system as new variants have emerged. However, because of the predilection for SARS-CoV-2 in causing impairments to the lungs and respiratory system, this study particularly focused on assessing the direct consequences to the respiratory system over the course of the pandemic by examining the frequency of the most common features related to the most dominant and prevalent SARS-CoV-2 variants. Additionally, this study sought to assess the effect of vaccination status on respiratory features. A few observations warrant additional discussion.

First, during the Delta and Omicron waves, there was a statistically significant difference in mortality between fully vaccinated and unvaccinated individuals. For partially vaccinated individuals, there was a significant reduction in mortality during the Alpha and Delta waves compared with individuals who were unvaccinated. Additionally, after combining individuals who either were fully vaccinated or partially vaccinated and comparing them with unvaccinated individuals, there were statistically significant reductions in mortality during the Delta and Omicron waves. The adjusted analyses showed that age and gender are confounding variables in the relationship between the independent variable (i.e. vaccination status) and the outcome variable (i.e. mortality). These findings also correspond to the findings reported in Tables 12 and 13, in which there were also significant differences in many prominent features such as pneumonia, acute respiratory failure, and hypoxemia between the fully vaccinated or partially vaccinated individuals and unvaccinated individuals.

Second, a major source of mortality of COVID-19 disease includes acute respiratory distress syndrome (ARDS) and respiratory failure. We show that as variants evolve there is a reduction in lower respiratory tract features, such as pneumonia, hypoxemia, and acute respiratory failure. This finding may be due to increasing rates of vaccination, a reduction in virulence with successive variants, improvement in management of care for patients with acute COVID-19 infection, acquisition of immunity among those reinfected or a combination of any of these factors. The drastic reduction in the lower respiratory symptoms of pneumonia across variants may best demonstrate this phenomenon- during the Founder phase, pneumonia was reported in 36.83 per 100 cases; however, during the Omicron phase, the frequency of pneumonia was reduced to 3.59 in fully vaccinated patients and 4.42 in unvaccinated individuals. Moreover, the statistically significant difference between the fully vaccinated, partially vaccinated, and unvaccinated patients in frequency of pneumonia supports the evidence regarding the immense benefits associated with vaccination in preventing severe disease. Although the reduction in lower respiratory features and increased mortality observed during the Delta period may appear contradictory, this is only evident if it is assumed that the individual died from ARDS; unfortunately, the UC CORDS data set does not contain information regarding the specific cause of death, so it is difficult to determine whether an individual’s cause of death was ARDS, some other related sequalae of COVID-19 infection (e.g. myocardial infarction), or some other pathology altogether.

Third, the respiratory features observed with each variant have evolved. In the current study we observed that the incidence of lower respiratory tract features decreased with successive emergence of variants, and that there was a concurrent increase in upper respiratory tract features. For example, Figs. 1 and 4 show a general decrease in frequencies for lower respiratory features with each successive variant. Meanwhile, for upper respiratory symptoms, Figs. 1 and 4 also show a more consistent pattern in overall frequency of upper respiratory features and furthermore, Figs. 2 and 5 show an uptick in the frequency of acute pharyngitis, acute upper respiratory infection, disorder of nasal cavity, and nasal congestion during the Delta and Omicron waves. Figures 3 and 6 depict the steep declines seen for all the lower respiratory features for successive variants of the COVID-19 pandemic. The findings are consistent with other studies, such as that by Wang et al.^3^, which showed that the Omicron variant was associated with less likelihood of 3-day risk of emergency department visit, hospitalization, intensive care unit admission, and mortality, because of Omicron being less virulent in causing lung-related disease^3^.

In contrast to the decreasing lower respiratory symptoms observed in the later SARS-CoV-2 variants, there was a notable increase in the trend of upper respiratory symptoms in this study. In particular, the features of acute pharyngitis, acute upper respiratory infection, and cough all either increased or remained elevated during more recent stages of the pandemic. These findings suggest that infection with more recent SARS-CoV-2 variants produces more upper airway features than lower airway features. More research is necessary to conclude that this is the case, but these findings are congruent with studies involving animals^17,18^.

### Limitations

This study is not without limitations. First, the nature of the retrospective design is biased towards those who either sought care or required hospitalization for COVID-19; thus, these data will not include information regarding patients who did not seek care. Second, this study did not account for the timing of when patients received vaccination and when they became ill with COVID-19. The possibility remains that patients may have received the full doses of the vaccine, but perhaps developed COVID-19 prior to the time required by their body to develop sufficient antibodies. Moreover, UC CORDS does not account for severity of illness when a patient tested positive for COVID-19, it is plausible that a patient may have tested positive for COVID-19 but had few symptoms or may have had many. Third, although the UC CORDS data set contains the records of over 2500 patients infected with the Founder variant, at this stage of the pandemic, the virus was still a novel phenomenon and the UC CORDS database had not yet been fully set up; therefore, the records of some of the patients infected with the Founder variant may be lacking or missing. Fourth, symptom selection was determined through expert identification of symptoms and corresponding ICD-10 code obtained from the electronic health record, so there may be diagnoses which existed but were not captured in the UC CORDS database. Moreover, both “pneumonia” and “viral pneumonia” ranked in the top 40 of feature collection, but due to similarities in presentation, “viral pneumonia” was combined with pneumonia, thus raising the possibility that the actual frequency of pneumonia may have been slightly lower than what was captured in this study due to potential overlap. Fifth, genomic data of the viral strains is unavailable in the UC CORDS data set, and therefore it was necessary to rely on the information from the CDC’s data tracker to determine time periods in which a particular strain was most likely to be dominant in the US; it is highly probable that the various strains overlapped as newer strains became more dominant. Sixth, there were likely some patients who developed re-infection resulting in their records to be counted twice leading to inconsistent total number of cases included in the study. Lastly, this study did not consider patients who may have had previous COVID-19 infection or having prior COVID-19 vaccination and a booster dose as having prior immunity; therefore, there may be patients in the not fully vaccinated group who retained some level of immunity related to previous COVID-19 infection.

### Future research

This study primarily focused on the most common respiratory features of patients with COVID-19 while controlling for vaccination status. Future studies should examine the frequency data while controlling for the timing of vaccine administration. Furthermore, because vaccination guidelines changed throughout the pandemic, it is necessary to re-examine the data while using the latest vaccine guidelines (e.g. fully vaccinated in this study was considered to be at least two doses; however, patients may have received an adenovirus vaccine, which initially only required one dose, while other may have received a mRNA vaccine, which initially required two doses). As guidelines continue to shift and the virus continues to mutate, future research should take into consideration the changing guidelines and definition of what “fully vaccinated” is considered to mean.

Additionally, as ongoing research demonstrates the significant long-term effects of COVID-19 infection on developing post-acute sequelae of SARS-CoV-2 infection (PASC), it is imperative to assess how acute COVID-19 infection manifests for patients in the long term. Of particular concern is of how the different variants may be associated with the development of PASC symptoms. Finally, as the COVID-19 pandemic continues to cause immense problems for patients and the healthcare system alike, it is essential to examine historical data to help inform present and future decision-making.

## Conclusion

This retrospective study examined the frequency of respiratory features across four major variants since the outset of the COVID-19 pandemic. Additionally, patients were categorized based on vaccination status and mortality risk was assessed. This study found that there were significant reductions in the risks of mortality for patients who were vaccinated during the Delta period. Additionally, there are substantially fewer lower respiratory features associated with later variants, such as Omicron. Meanwhile, as the frequency of lower respiratory features has decreased, there is a substantial uptick in the frequency of upper respiratory features. This study also showed substantial favorable benefits in patients who are fully vaccinated compared with the unvaccinated or only partially vaccinated, the fully vaccinated population experienced significantly fewer features involving the upper and lower respiratory tract. This study indicates that because of numerous factors, including viral evolution, enhanced immunity, and likely improved treatment modalities, respiratory features involving the lower respiratory tract are reported with less frequency compared with earlier stages of the pandemic.

## Supplementary Information

Supplementary Information.

## Data Availability

The data set used in this study, University of California Covid Research Data Set (UC CORDS), is made available by the University of California Office of the President and the University of California Biomedical, Research, Acceleration, Integration, and Development. The data set analyzed during this study is not publicly available. Questions regarding the UC CORDS Data Set can be directed to the project contact, Atul Butte (atul.butte@ucsf.edu).
