# Supplementary material for: The effect of SARS-CoV-2 variant on respiratory features and mortality
Source: Sci Rep. 2023 Mar 18;13:4503. doi: 10.1038/s41598-023-31761-y (PMC10024278; doi:10.1038/s41598-023-31761-y)
Supplement: Supplementary file 1 — Supplementary Information. [file 41598_2023_31761_MOESM1_ESM.docx]

**The Effect of SARS-COV-2 Variant on Respiratory Features and Mortality**

Thomas D. Hughes^1*^ (thomasdh@hs.uci.edu), Ajan Subramanian^1*^ (ajans1@uci.edu), Rana Chakraborty^2^ (Chakraborty.Rana@mayo.edu), Shannon A. Cotton^1^ (sacotton@uci.edu), Maria Del Pilar Giraldo Herrera^1^ (mdgirald@uci.edu), Yong Huang^1^(yongh7@uci.edu), Natalie Lambert^3^ (nalamb@iu.edu), Melissa D. Pinto^1^ (mdpinto@hs.uci.edu), Amir M. Rahmani^1^ (a.rahmani@uci.edu), Carmen Josefa Sierra^4^ (carmenjsierra@miami.edu), & Charles A. Downs^4⤉^ (cxd826@miami.edu)

* Authors contributed equally to warrant co-first authors

^1^ University of California, Irvine, ^2^ Mayo Clinic, ^3^ Indiana University, ^4^ University of Miami, ^⤉^ Corresponding author

**Supplementary Information Figures and Tables**


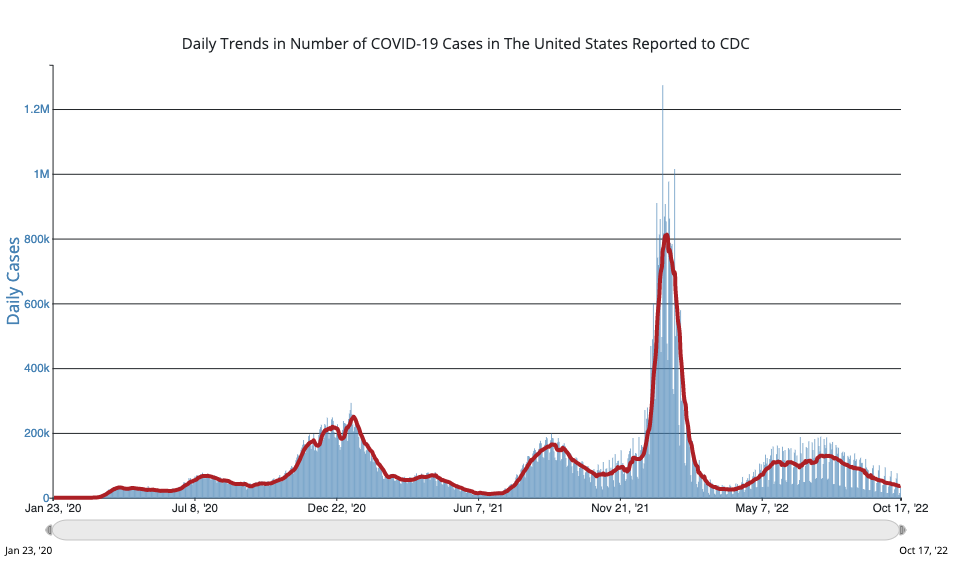


**Supplementary Figure 1**

Daily trends in number of COVID-19 cases in the United States reported to the CDC [2].


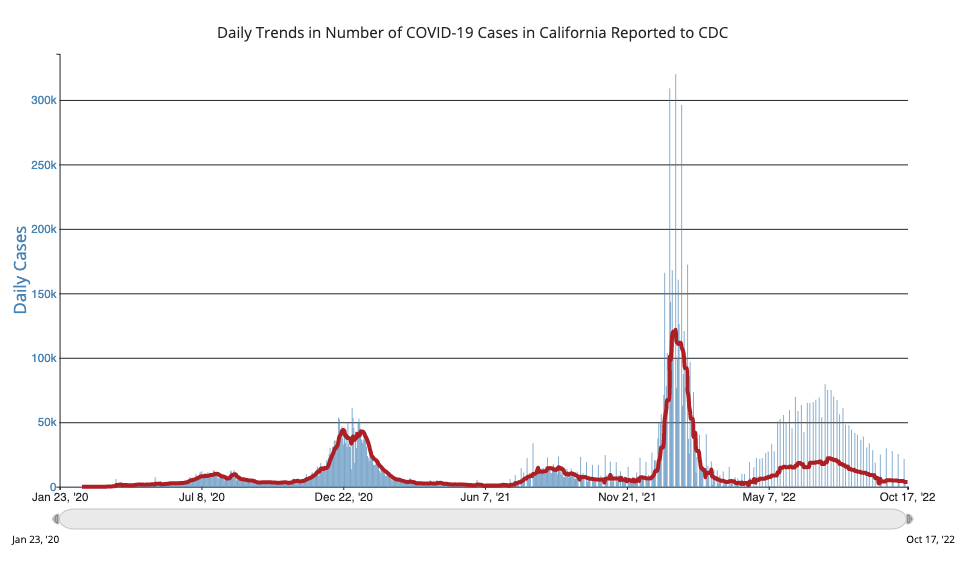


**Supplementary Figure 2**

Daily trends in number of COVID-19 cases in California reported to the CDC [14].

| **Rank** | **Feature** | **Classification** |
| --- | --- | --- |
| 1 | Pneumonia | Lower respiratory |
| 2 | Cough | Upper respiratory |
| 3 | Fever | Unclassified |
| 4 | Essential hypertension | Unclassified |
| 5 | Acute respiratory failure | Lower respiratory |
| 6 | Type 2 diabetes mellitus | Unclassified |
| 7 | Dyspnea | Lower respiratory |
| 8 | Sepsis | Unclassified |
| 9 | Hyperlipidemia | Unclassified |
| 10 | Acute upper respiratory infection | Upper respiratory |
| 11 | Acute renal failure | Unclassified |
| 12 | Hyponatremia | Unclassified |
| 13 | Hypoxemia | Lower respiratory |
| 14 | Viral disease | Unclassified |
| 15 | Diarrhea | Unclassified |
| 16 | Anemia | Unclassified |
| 17 | Septic shock | Unclassified |
| 18 | Gastroesophageal reflux with esophagitis | Unclassified |
| 19 | Upper respiratory tract infection due to Influenza | Upper respiratory |
| 20 | Obesity | Unclassified |
| 21 | Hyperglycemia | Unclassified |
| 22 | Acidosis | Unclassified |
| 23 | Reduced mobility | Unclassified |
| 24 | Abnormal lung findings | Lower respiratory |
| 25 | Acute respiratory distress syndrome | Lower respiratory |
| 26 | Headache | Unclassified |
| 27 | Chest pain | Unclassified |

**Supplementary Table 1**

Feature rank, feature, and classification of features of the Founder variant.

| **Rank** | **Feature** | **Classification** |
| --- | --- | --- |
| 1 | Pneumonia | Lower respiratory |
| 2 | Essential hypertension | Unclassified |
| 3 | Hyperglycemia in diabetes | Unclassified |
| 4 | Type 2 diabetes mellitus | Unclassified |
| 5 | Acute respiratory failure | Lower respiratory |
| 6 | Fever | Unclassified |
| 7 | Hyperlipidemia | Unclassified |
| 8 | Dyspnea | Lower respiratory |
| 9 | Sepsis | Unclassified |
| 10 | Acute renal failure | Unclassified |
| 11 | Hyponatremia | Unclassified |
| 12 | Reduced mobility | Unclassified |
| 13 | Hypoxemia | Lower respiratory |
| 14 | Anemia | Unclassified |
| 15 | Gastroesophageal reflux without esophagitis | Unclassified |
| 16 | Hyperglycemia in diabetes | Unclassified |
| 17 | Acidosis | Unclassified |
| 18 | Headache | Unclassified |
| 19 | Chest pain | Unclassified |
| 20 | Acute pharyngitis | Upper respiratory |
| 21 | Acute upper respiratory infection | Upper respiratory |
| 22 | Diarrhea | Unclassified |
| 23 | Viral disease | Unclassified |
| 24 | Fatigue | Unclassified |
| 25 | Disorder of nasal cavity | Upper respiratory |
| 26 | Tachycardia | Unclassified |
| 27 | Abnormal lung findings | Lower respiratory |
| 28 | Acquired absence of organ | Unclassified |
| 29 | Hyperglycemia not due to diabetes | Unclassified |
| 30 | Immunodeficiency disorder | Unclassified |

**Supplementary Table 2**

Feature rank, feature, and classification of features of the Alpha variant.

| **Rank** | **Feature** | **Classification** |
| --- | --- | --- |
| 1 | Pneumonia | Lower respiratory |
| 2 | Cough | Upper respiratory |
| 3 | Essential hypertension | Unclassified |
| 4 | Fever | Unclassified |
| 5 | Acute respiratory failure | Lower respiratory |
| 6 | Type 2 diabetes mellitus | Unclassified |
| 7 | Acute upper respiratory infection | Upper respiratory |
| 8 | Dyspnea | Lower respiratory |
| 9 | Hyperlipidemia | Unclassified |
| 10 | Sepsis | Unclassified |
| 11 | Acute renal failure | Unclassified |
| 12 | Acute pharyngitis | Upper respiratory |
| 13 | Disorder of nasal cavity | Upper respiratory |
| 14 | Viral disease | Unclassified |
| 15 | Hyponatremia | Unclassified |
| 16 | Headache | Unclassified |
| 17 | Gastroesophageal reflux without esophagitis | Unclassified |
| 18 | Obesity | Unclassified |
| 19 | Hypoxemia | Lower respiratory |
| 20 | Chest pain | Unclassified |
| 21 | Anemia | Unclassified |
| 22 | Fatigue | Unclassified |
| 23 | Diarrhea | Unclassified |
| 24 | Nasal congestion | Upper respiratory |
| 25 | Reduced mobility | Unclassified |
| 26 | Tachycardia | Unclassified |
| 27 | Acute respiratory distress syndrome | Lower respiratory |
| 28 | Acidosis | Unclassified |
| 29 | Hyperglycemia not due to diabetes | Unclassified |
| 30 | Kidney transplant present | Unclassified |
| 31 | Abnormal lung findings | Lower respiratory |
| 32 | Upper respiratory tract infection due to Influenza | Upper respiratory |
| 33 | Acquired absence of organ | Unclassified |
| 34 | HIV infection | Unclassified |

**Supplementary Table 3**

Feature rank, feature, and classification of features of the Delta variant.

| **Rank** | **Feature** | **Classification** |
| --- | --- | --- |
| 1 | Cough | Upper respiratory |
| 2 | Essential hypertension | Unclassified |
| 3 | Type 2 diabetes mellitus | Unclassified |
| 4 | Fever | Unclassified |
| 5 | Acute upper respiratory infection | Upper respiratory |
| 6 | Acute pharyngitis | Upper respiratory |
| 7 | Hyperlipidemia | Unclassified |
| 8 | Pneumonia | Lower respiratory |
| 9 | Dyspnea | Lower respiratory |
| 10 | Disorder of nasal cavity | Upper respiratory |
| 11 | Gastroesophageal reflux without esophagitis | Unclassified |
| 12 | Acute respiratory failure | Lower respiratory |
| 13 | Immunodeficiency disorder | Unclassified |
| 14 | Viral disease | Unclassified |
| 15 | Acute renal failure syndrome | Unclassified |
| 16 | Chest pain | Unclassified |
| 17 | Anemia | Unclassified |
| 18 | Headache | Unclassified |
| 19 | Acquired absence of organ | Unclassified |
| 20 | Sepsis | Unclassified |
| 21 | Obesity | Unclassified |
| 22 | Hyponatremia | Unclassified |
| 23 | Nasal congestion | Upper respiratory |
| 24 | Fatigue | Unclassified |
| 25 | Pregnant | Unclassified |
| 26 | Reduced mobility | Unclassified |
| 27 | Tachycardia | Unclassified |
| 28 | Diarrhea | Unclassified |
| 29 | Kidney transplant present | Unclassified |
| 30 | Hyperglycemia due to diabetes | Unclassified |
| 31 | Upper respiratory tract infection due to Influenza | Upper respiratory |
| 32 | Pain | Unclassified |

**Supplementary Table 4**

Feature rank, feature, and classification of features of the Omicron variant.
